# Haemodynamic Adaptive Mechanisms at High Altitude: Comparison between European Lowlanders and Nepalese Highlanders

**DOI:** 10.3390/jcm11133843

**Published:** 2022-07-02

**Authors:** Paolo Salvi, Andrea Grillo, Sylvie Gautier, Luca Montaguti, Fausto Brunacci, Francesca Severi, Lucia Salvi, Enzo Pretolani, Gianfranco Parati, Athanase Benetos

**Affiliations:** 1 Department of Cardiology, Istituto Auxologico Italiano, IRCCS, 20149 Milan, Italy; gianfranco.parati@unimib.it; 2 Medicina Clinica, Azienda Sanitaria Universitaria Giuliano Isontina, 34149 Trieste, Italy; andr.grillo@gmail.com; 3 CHRU-Nancy, Pôle “Maladies du Vieillissement, Gérontologie et Soins Palliatifs”, Université de Lorraine, 54000 Nancy, France; s.gautier@chru-nancy.fr (S.G.); a.benetos@chru-nancy.fr (A.B.); 4 Department of Internal Medicine, ‘M. Bufalini’ Hospital, AUSL Romagna, 47521 Cesena, Italy; luca.montaguti@auslromagna.it (L.M.); fausto.brunacci@libero.it (F.B.); francescaseveri777@gmail.com (F.S.); psalvi.md@libero.it (E.P.); 5 Medicina II Cardiovascolare, AUSL-IRCCS di Reggio Emilia, 42123 Reggio Emilia, Italy; lsalvi.md@gmail.com; 6 Department of Medicine and Surgery, University of Milano-Bicocca, 20126 Milan, Italy; 7 INSERM, Université de Lorraine, DCAC u1116, 54000 Nancy, France

**Keywords:** altitude, altitude sickness, aortic stiffness, aortic distensibility, atrial natriuretic factor, blood pressure, pulse wave velocity, vascular stiffness

## Abstract

Background: Exposure to high altitudes determines several adaptive mechanisms affecting in a complex way the whole cardiovascular, respiratory, endocrine systems because of the hypobaric hypoxic condition. The aim of our study was to evaluate the circulatory adaptive mechanisms at high altitudes, during a scientific expedition in the Himalayas. Methods: Arterial distensibility was assessed measuring carotid-radial and carotid-femoral pulse wave velocity. Tests were carried out at several altitudes, from 1350 to 5050 m above sea level, on 8 lowlander European researchers and 11 highlander Nepalese porters. Results: In Europeans, systolic blood pressure and pulse pressure increased slightly but significantly with altitude (*p* < 0.05 and *p* < 0.001, respectively). Norepinephrine showed a significant increase after the lowlanders had spent some time at high altitude (*p* < 0.001). With increasing altitude, a progressive increase in carotid-radial and carotid-femoral pulse wave velocity values was observed in lowlanders, showing a particularly significant increase (*p* < 0.001) after staying at high altitude (carotid-radial pulse wave velocity, median value (interquartile range) from 9.2 (7.9–10.0) to 11.2 (10.9–11.8) m/s and carotid-femoral pulse wave velocity from 8.5 (7.9–9.0) to 11.3 (10.9–11.8) m/s). At high altitudes (3400 and 5050 m above sea level), no significant differences were observed between highlanders and lowlanders in hemodynamic parameters (blood pressure, carotid-radial and carotid-femoral pulse wave velocity). Conclusions: The progressive arterial stiffening with altitude observed in European lowlanders could explain the increase in systolic and pulse pressure values observed at high altitudes in this ethnic group. Further studies are needed to evaluate the role of aortic stiffening in the pathogenesis of acute mountain sickness.

## 1. Introduction

Exposure to high altitudes determines several adaptive mechanisms which affect in a complex way the whole cardiovascular, respiratory, and endocrine systems because of the hypobaric hypoxic condition. Altitude may lead to detrimental effects on health, the most common of which is Acute Mountain Sickness (AMS). This condition is favoured by rapid ascent, exercise, cold, and individual predisposition [1,2] and is characterized by a clinical syndrome which may result in pulmonary and cerebral oedema, which are potentially lethal events [3,4]. The interrelation of several haemodynamic factors contributes to the development of pulmonary oedema. With the scope to evaluate the role of cardiovascular alterations in the pathogenesis of high-altitude pulmonary oedema, several studies have been conducted on humans to study the pulmonary and cardiac adaptations to high altitudes [5]. The adrenergic system activation is particularly important for the control of the cardiac function and of the vascular tone in the first phases of high altitudes adaptation [6]. In men, this activation seems to be particularly evident during a sub maximal physical stress [7] and could be responsible for the increased values of the basal renin plasmatic activity observed in these individuals [8]. Further studies have also demonstrated a modification in the number and functions of beta-adrenergic receptors in highlanders [9]. In the critical conditions of high altitudes, some human populations, such as Nepalese highlanders, have developed some phenotypic traits influenced by hypoxia, namely, a broader chest, larger lung capacity, and increase haemoglobin concentration [10,11,12]. The biological response of healthy lowlanders exposed to high altitude may therefore differ from that of native highlanders.

Despite the large number of studies conducted on humans to study the pulmonary and cardiac adaptations to altitude, the adaptive mechanisms of the large elastic arteries at very high altitude are not yet clearly understood. Given the difficulty and complexity of performing clinical research in extreme hypoxic-hypobaric conditions, a very small number of studies evaluated a possible process of aortic stiffening induced by exposure at very high altitudes. Lewis et al. [13] assessed arterial viscoelastic properties on 12 healthy lowlanders and 12 highlanders at very high altitude. In this study aortic distensibility, estimated by carotid-femoral pulse wave velocity (PWV), was measured in lowlanders at sea level, upon arrival at 5050 m above sea level (a.s.l.), and after 12–14 days of acclimatization, while highlanders completed only one session at 5050 m a.s.l. Compared with lowlanders at sea level, highlanders showed a higher aortic PWV; however, once lowlanders were exposed to high altitude, these between group differences were not present. However, the small number of people involved in the study and the absence of assessments at intermediate altitudes require further studies to confirm these results. On the other hand, acute ascent at an altitude of 4559 m a.s.l. (HIGHCARE Alps Study, Capanna Regina Margherita, Monte Rosa, Italy) did not show a significant change in aortic PWV in a group of 22 healthy volunteers [14].

The large arteries play an important role in blood pressure and peripheral flux regulation. It is well known that large arteries physiologically have not only a conduit function but also a buffering function, and owing to their distensibility, they are able to decrease the pulsatile systolic output of the left ventricle. Therefore, the large arteries have a regulation role, redeeming the pulsatility of the systolic ejection and transforming the regime of the cardiac pump from discontinuous to continuous. This buffering function results from the viscoelastic properties of the arterial wall, which depend on the arterial structure and tone. Structure is determined by the three arterial wall components: elastin, collagen, and smooth muscle cells. On the other hand, the arterial tone is mainly modulated by the autonomic nervous system’s activity and by other vasoactive systems (adrenergic system, renin-angiotensin system, vasopressin, etc.) [15]. The functional and structural conditions of the arteries determine their ability to buffer the systolic wave and influence systemic blood pressure values [16].

The aim of our research was to study the changes in blood pressure and haemodynamic parameters during the ascent and staying at high altitudes, in two groups of European lowlanders and Nepalese highlanders.

## 2. Materials and Methods

This study was performed during the scientific expedition in the Himalayas “Circulatory adaptive mechanisms at high altitudes”, inside the research project Everest-K2 of the Italian National Research Council (CNR). Our scientific expedition involved a group of white European lowlander researchers, who lived permanently almost at sea level, and a group of Nepalese highlanders, of Rai ethnicity, born and residing in the Khumbu valley between 3400 and 4930 m a.s.l., on average at 4007 ± 583 m a.s.l. The presence of chronic disease involving habitual therapy was considered an exclusion criterion from the study. No chronic treatments were taken by the study participants, and no drugs were taken during the high-altitude ascension.

The lowlanders, coming from Europe (Milan and Paris), after a 3-day stay in Kathmandu (Nepal, 1350 m a.s.l.), were taken by air transport to Lukla airport (2840 m a.s.l.). Nepalese highlander porters were enrolled at Lukla airport. Enrolment was random, linked to the needs of the scientific expedition (transport of scientific instruments and personal baggage of the researchers). After staying overnight in the nearby village of Phakding (2500 m a.s.l.), the group of lowlanders and highlanders trekked the next day to the village of Namche Bazaar (3400 m a.s.l.), where they stayed two full days for ac-climatization. From Namche Bazaar, the two groups of lowlanders and highlanders hiked together for 3 days to Lobuche (5050 m a.s.l.) on the Nepalese side of Everest at the “Pyramid International Laboratory” of the Italian National Research Council (CNR).

Data were collected at Kathmandu and Namche Bazaar in hotel rooms and at Lobuche in the Pyramid International Laboratory. Barometric pressure was recorded by a microclimatic station at the time of each study. Ambient temperature was similarly recorded and kept constant throughout the study. Examinations were performed in the morning after at least 18 h of physical rest and after a stay of at least 1 h in a room with a constant temperature of 19 ± 1 °C.

### 2.1. Protocol of the Study

Participants were studied at different altitudes.

**Figure 1:**
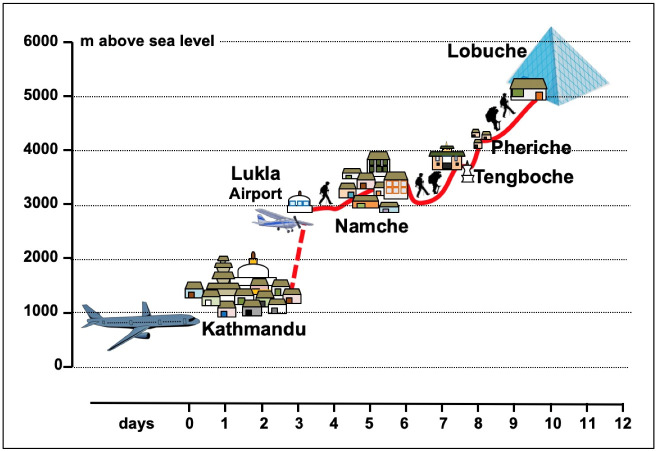
Timing, altimetry, and steps of the scientific expedition.

For the Europeans, measurements were performed (Figure 1):

- In Italy, at sea level (only clinical and blood parameters);
- On the second day of permanence in Kathmandu, at 1350 m a.s.l.;
- On the second day of permanence in Namche Bazar, at 3400 m a.s.l.;
- On the second day of permanence in Lobuche, at 5050 m. a.s.l.;
- On the eighth day of stay in this high altitude laboratory.

For Nepalese participants, the same measurements were performed the same day as for the Europeans at 3400 m a.s.l. and the second day after their arrival in Lobuche.

### 2.2. Arterial Stiffness Assessment

The viscoelastic properties of large arteries were estimated by measuring PWV. Currently, carotid-femoral PWV (cf-PWV) is considered the most reliable non-invasive method for assessing aortic stiffness, while carotid-radial PWV (cr-PWV) reflects the stiffness of the muscular arteries in the upper limb [17]. A Millar Mikro-Tip Pulse Transducer SPT-301B tonometer (Millar Instruments, Inc., Houston, TX, USA) [16,18,19] integrated into a Cardioline Delta 3 electrocardiograph (Remco SpA, San Pedrino di Vignate, Italy) was used to record pulse pressure curves. PWV was measured by recording pressure wave curves in the carotid and peripheral arteries (femoral or radial) in rapid succession. PWV was defined as 80% of the distance between measurement sites [20] divided by the time delay between the distal (femoral or radial) pulse wave and the proximal (carotid) pulse wave, using the ECG trace as a reference. This method was previously described in detail [21,22].

### 2.3. Blood Pressure Measurement

Blood pressure measurements were carried out by means of mercury sphygmomanometer and a validated oscillometric system (Dinamap, model 1846 SX, Critikon, Tampa, FL, USA). Blood pressure with the latter device was acquired every 2 min in the left arm, during the tonometric recording.

### 2.4. Oxygen Saturation

Arterial oxygen saturation was measured by means of a Kontron Pulse Oximeter 7845 (Kontron, S&T group, Linz, Austria) with finger clip sensor.

### 2.5. Biochemical Dosages

The radioenzymatic assay was performed for the dopamine, epinephrine, and norepinephrine assays and the radioimmunoassay for the determination of the atrial natriuretic factor. These dosages were performed only at 1350 m a.s.l., at 5050 m a.s.l., and after stay at 5050 m a.s.l.

### 2.6. Statistical Analysis

Results are expressed as median and interquartile range. Normal distribution of variables was assessed by Shapiro–Wilk test. Differences between two groups (Europeans and Nepalese) for all variables were evaluated with Student’s *t*-test for unpaired data or with independent samples Mann–Whitney U test for variables not normally distributed. Levene’s test was used to assess equality of variances. Statistical analysis of parameter’s alterations with altitude was calculated using Friedman’s test and the subsequent two tailed Wilcoxon test for non-parametric paired data. Multivariate analysis using logistic regression models adjusted for age and body mass index were performed to assess differences in parameters between the European and Nepalese groups. Statistical analysis was performed by using the Statistical Package for the Social Sciences (SPSS for Windows, Release 20.0; SPSS, Chicago, IL, USA). A *p* value less than 0.05 was considered as significant.

## 3. Results

### 3.1. Population

All 8 European participants in the scientific expedition (6 men and 2 women) and 11 male Nepalese porters belonging to the Rai ethnic group agreed to participate in the study. Table 1 shows how highlander Nepalese were significantly shorter and leaner than European lowlanders.

Among the eight Europeans participating in this expedition, six reported headaches after 4000 m a.s.l. Three had moderate dyspnoea at rest, and one experienced vomiting at 5000 m a.s.l. Finally, upon arrival at Lobuche, one person in the lowlander group presented with signs of moderate cerebral oedema (ataxia, headache, dizziness, vomiting), which rapidly regressed after oxygen and corticoid therapy. None of the highlander porters reported any symptoms.

### 3.2. Blood Pressure and Large Arteries Parameters

Table 2 shows the clinical and haemodynamic parameters changes with altitude in the lowlander European volunteers and in the Nepalese porters. 

**Figure 2:**
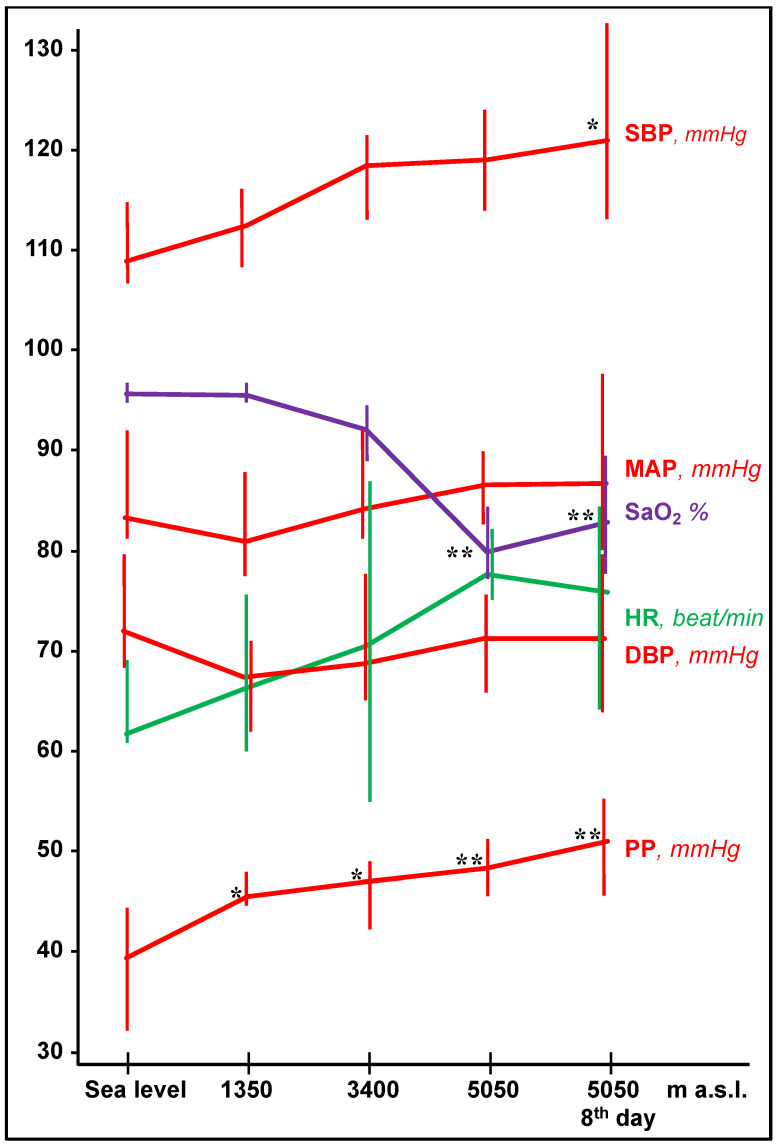
Heart rate (HR, green line), arterial oxygen saturation (SaO_2_, violet line), and blood pressure (red lines) changes with altitude in 8 lowlander European volunteers. Data are shown as median and interquartile range. Significance is expressed by the *p*-value: *, *p* < 0.05; **, *p* < 0.001 versus sea level values. a.s.l., above sea level; DBP, diastolic blood pressure; MAP, mean arterial pressure; PP, pulse pressure (= SBP − DBP); SBP, systolic blood pressure.

In the European group, systolic blood pressure and pulse pressure increased slightly but significantly with altitude (Figure 2), reaching the highest levels eight days after the arrival at 5050 m (*p* < 0.05). Diastolic blood pressure and mean blood pressure did not show significant changes with altitude. In the Nepalese participants, blood pressure values did not change between 3500 and 5050 m.

**Figure 3:**
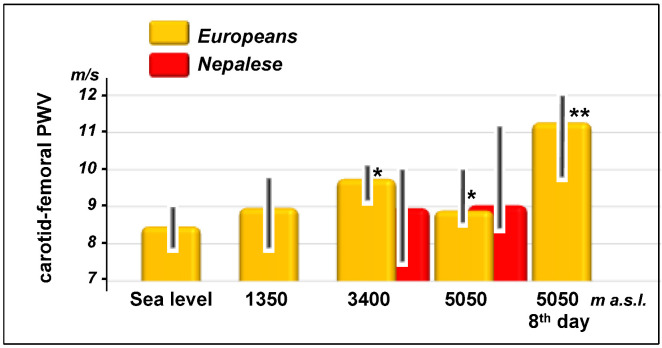
Changes in carotid-femoral pulse wave velocity (PWV) during the ascent and stay at very high altitudes in Europeans and Nepalese. Data are shown as median and interquartile range. Significance versus basal condition (sea level for lowlanders and 3400 m a.s.l. for highlanders) is expressed by the *p*-value: *, *p* < 0.05; **, *p* < 0.001.

**Figure 4:**
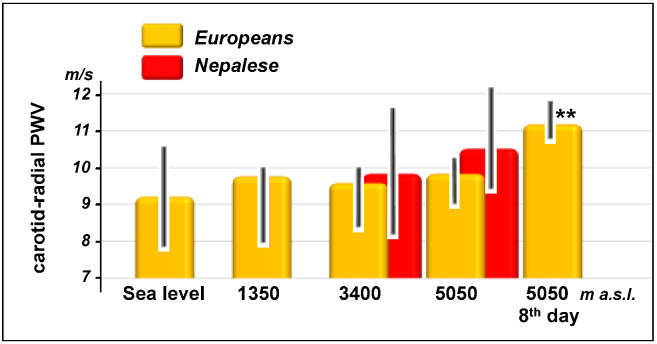
Changes in carotid-radial pulse wave velocity (PWV) during the ascent and stay at very high altitudes in Europeans and Nepalese. Data are shown as median and interquartile range. Significance versus basal condition (sea level for lowlanders and 3400 m a.s.l. for highlanders) is expressed by the *p*-value: **, *p* < 0.001.

Carotid-femoral and carotid-radial PWV increased in Europeans with altitude (Figure 3 and Figure 4) showing the highest significant values (*p* < 0.001) on the eighth day in Lobuche (carotid-femoral: from 8.5 (7.9–9.0) to 11.3 (10.9–11.8) m/s, carotid-radial: from 9.2 (7.9–10.0) to 11.2 (10.9–11.8) m/s). As for systolic blood pressure in the Nepalese, no changes in PWV were observed with altitude.

**Figure 5:**
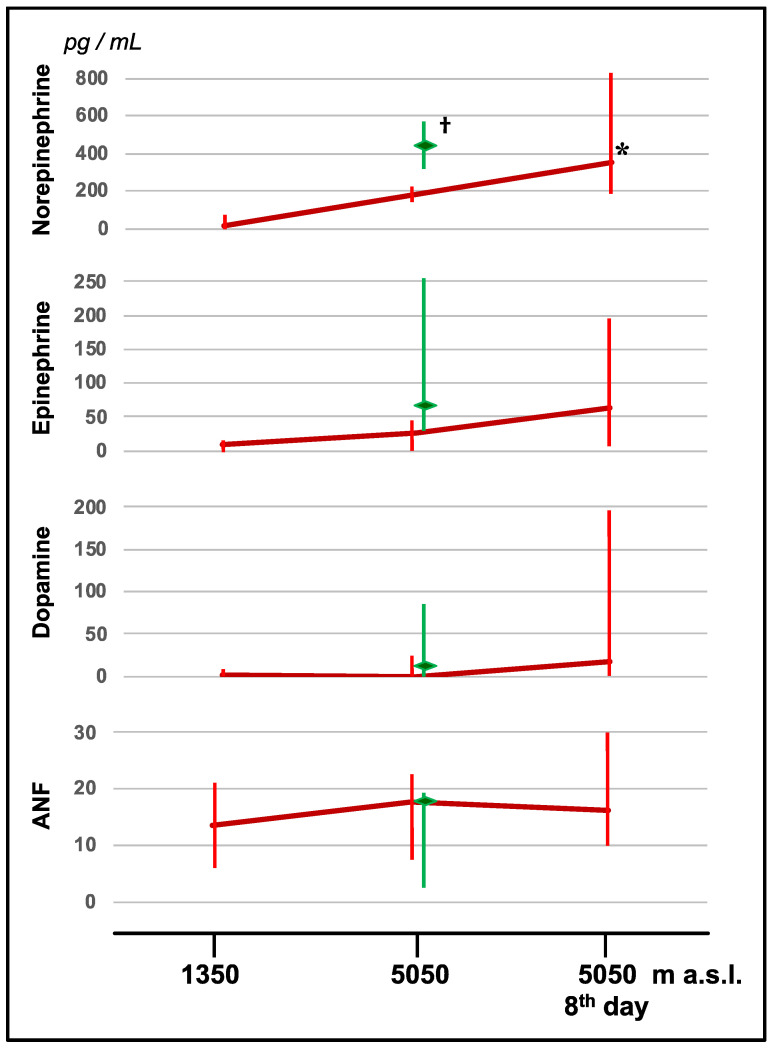
Changes in norepinephrine, epinephrine, dopamine, and atrial natriuretic factor values in lowlanders (red lines) with altitude. Data recorded in highlanders at 5050 m a.s.l. are shown as green lines. Data are shown as median and interquartile range. a.s.l., above sea level. Significance is expressed by the *p*-value: *, *p* < 0.05 versus values at 1350 m a.s.l.; †, *p* < 0.05 highlanders versus lowlanders.

A constant elevation of norepinephrine was observed in lowlanders at high altitude (5050 m a.s.l.), especially on the eighth day compared to the values recorded at 1350 m a.s.l. (*p* < 0.05): from median (interquartile range) 18 (9–45) pg/mL at 1350 m a.s.l. to 186 (158–222) pg/mL at 5050 m a.s.l., to 350 (193–833) pg/mL after 8 days of stay at 5050 m a.s.l. Nepalese people had higher levels of norepinephrine than the Europeans at the same altitude (428 (326–572) pg/mL at 5050 m a.s.l., *p* < 0.05). Epinephrine, dopamine, and atrial natriuretic factor tended to increase with altitude in lowlanders, without reaching statistical significance (Figure 5). No significant differences were found in the dosages of these molecules between lowlanders and highlanders at 5050 m a.s.l.

## 4. Discussion

Our study provides three main findings. One, in healthy lowlander volunteers, acute exposure to high altitude causes a significant increase in arterial stiffness, as documented by the increase in PWV. Two, this process of stiffening of the large arteries not only affects the aorta but also involves the large muscular arteries of the upper limbs. Three, PWV values (both carotid-femoral and carotid-radial) recorded in Nepalese highlanders were similar to those recorded in European lowlanders at the same altitude. These changes remained significant after adjusting for mean arterial pressure and heart rate changes with altitude.

The viscoelastic properties of the aorta and of the large elastic arteries are guaranteed by an adequate ratio between elastic and collagen fibres of the arterial wall, as well as by a balance between these fibres, the extracellular matrix, and the smooth muscle cells. While changes in arterial wall structure may be evoked as an adaptation phenomenon for Nepalese highlanders, on the other hand, it is difficult to hypothesize that structural changes in the arterial wall can occur in the relatively short time of high-altitude ascension. A further element that makes PWV change at high altitude unlikely to be due to structural changes in the arterial wall comes from the results of the HIGHCARE Study. In agreement with results of our study, the HIGHCARE study showed, after an increase in PWV values at high altitudes, a rapid return of the PWV to baseline after returning to Europe at sea level [23].

We can therefore hypothesize, at least in lowlanders, a prevalent role played by functional factors in the changes in arterial distensibility at high altitudes. The role of functional factors affecting arterial mechanical properties is complex, and their changes are transitory. The main functional factors determining changes in vascular distensibility include left ventricular systolic ejection function, heart rate, arterial smooth muscle, and mean arterial pressure [16]. The sympathetic nervous system is considered to be one of the major elements affecting arterial functional properties. The activation of the sympathetic nervous system increases heart rate, ventricular contractility, and modulates the activity of the smooth muscle cell of the arterial wall, inducing peripheral vasoconstriction, increase in peripheral vascular resistance, and, therefore, a rise in mean arterial pressure. Additionally, increased peripheral vascular resistance can modify the amplitude and distribution of reflected pressure waves, increasing aortic systolic blood pressure as well as pulse pressure.

As already emerged in the HIGHCARE Study [23,24], and as shown by other research at high altitude [25,26], we confirme that exposure to high-altitude hypoxia is accompanied by a significant increase in plasma norepinephrine proportional to the altitude reached. This sympathetic activation is accentuated during the stay at high altitude. The adrenergic system activation is particularly important for the control of cardiac function and vascular tone in the first phases of adaptation to high altitude [6,24]. A modification of the number and functions of beta-adrenergic receptors in subjects who live at a high altitudes has been clearly shown [9]. Indeed, elevated epinephrine values at high altitudes were also found in our study, even in highlander porters.

The trend of the norepinephrine increase curve is associated with a corresponding significant increase in PWV. The stiffening of the aorta at high altitudes, documented by the increase in cf-PWV, may justify the increase in systolic blood pressure and pulse pressure recorded at high altitudes. The PWV values which were registered in the native people are similar to those registered in the Europeans at the same altitude; therefore, it may be inferred that the modifications which were observed in the Europeans are chronically present in the natives.

Muscular arteries should be much more sensitive to the activity of the sympathetic nervous system than the aorta and elastic arteries. We would therefore have expected a greater increase in cr-PWV compared to cf-PWV at high altitudes. Therefore, the weaker increase in carotid-radial PWV with altitude suggests that other factors besides sympathetic activation may influence the aortic stiffening. Among other factors that can contribute to increased aortic stiffness at high altitudes, we can consider haemoconcentration with consequent increase in blood viscosity [27], oxidative stress [28,29], interstitial oedema of the arterial wall [30], and endothelial dysfunction [31,32]. Previous studies have shown a link between altitude-induced increases in pulmonary artery pressure and increases in plasma and urinary tract endothelin-1 levels [33], suggesting that acute exposure to high altitude may impair both endothelial and vascular smooth muscle cell function. At present all these etiopathogenetic hypotheses are mainly based on speculative considerations, and further experimental studies are necessary to define the mechanisms underlying the increase in arterial stiffness related to hypobaric-hypoxia.

According to our knowledge, only the study developed by Lewis et al. compared aortic PWV values in lowlanders and highlanders at the same altitude, above 5000 m a.s.l., and provided evidence of impaired vascular function in highlanders versus lowlanders at sea level, as indicated by significantly higher central PWV values [13]. These observed changes in vascular function and central PWV in the Nepalese natives were remarkably comparable to those of European lowlanders at 5050 m a.s.l., suggesting that these changes may not depend on time spent at high altitudes. Contrary to what was evidenced in our study and in Lewis’s study, Bruno et al. found no differences in aortic PWV values when comparing a cohort of 95 Nepalese living permanently in three rural villages at 2600, 3800, and 3800 m a.s.l. with a group of 64 Caucasian Italian volunteers, matched for age, sex, mean arterial pressure, and body mass index [34]. The discrepancy between the results of these studies could be attributed to a selection bias in the lowlander control group of the latter study. Further studies are needed to clarify this issue.

The main limitation of our study was the relatively small number of enrolled individuals. However, the limit conditions in which the researchers worked must be taken into account. Materials were carried on back by Nepalese porters and yaks (therefore, the weight was limited to small amounts), the energy supply was scarce. In Namche, the electric power was available only for few hours a day and sometimes it was unsteady.

## 5. Conclusions

These data demonstrate how altitude determines important and significant haemodynamic changes. The stiffening of the aorta at high altitudes, documented by the increase in cf-PWV, could justify the increase in systolic blood pressure and pulse pressure recorded at high altitudes [23,35]. Further studies are needed to evaluate the role of aortic stiffening in the pathogenesis of high-altitude pulmonary oedema and of the cardiac hypertrophy-dilation, which is often observed in people exposed to prolonged stays at high altitudes and in highlanders affected by chronic mountain sickness.

## Figures and Tables

**Table 1:** Main anthropometric characteristics of participants.

Parameter	Lowlanders	Highlanders	*p*-Value
Sex, f/m	2/6	0/11	
Age, years	35.0 (1.5–37.7)	25.0 (23.0–33.0)	0.004
Height, cm	176.0 (167.0–178.7)	158.0 (155.0–166.0)	<0.001
Weight, Kg	76.5 (70.5–82.2)	51.0 (49.0–54.0)	<0.001
BMI, Kg/m^2^	25.0 (23.9–27.5)	20.6 (18.5–21.5)	<0.001
BSA, m^2^	1.93 (1.81–2.01)	1.50 (1.47–1.56)	<0.001

Data are shown as median (interquartile range). Significance is expressed by the *p*-value. BMI, body mass index; BSA, body surface area; f, females; m, males.

**Table 2:** Clinical and haemodynamic parameters changes with altitude in lowlander European volunteers (*n* = 8) and in highlander Nepalese porters (*n* = 11).

European Lowlanders					
Parameters	Sea Level	1350 m a.s.l.	3400 m a.s.l.	5050 m a.s.l.	5050 m a.s.l. after 8 Days of Stay
SaO_2_, %	96.0 (95.2–97.0)	95.9 (95.4–97.0)	92.6 (89.0–94.5)	80.5 (77.0–84.0) **	83.5 (77.8–89.5) **
Respiratory Rate, breaths/m	10.5 (10.0–11.0)	10.0 (10.0–10.7)	12.0 (11.2–13.7)	14.0 (12.2–15.0) *	14.0 (12.3–15.0) *
Heart Rate, beat/m	62.6 (62.0–69.3)	67.2 (59.8–74.9)	71.2 (54.8–86.7)	78.3 (74.6–82.4)	76.5 (63.8–83.7)
Systolic BP, mmHg	109.2 (107.7–115.2)	112.7 (109.1–116.4)	118.5 (112.2–120.9)	119.2 (114.5–122.2)	121.0 (111.5–133.0) *
Diastolic BP, mmHg	71.3 (68.0–79.5)	66.7 (62.6–71.3)	68.0 (64.7–77.1)	70.5 (66.2–75.6)	70.5 (63.1–80.2)
Mean BP, mmHg	84.0 (81.2–93.7)	81.6 (78.6–87.1)	84.8 (81.4–91.8)	87.2 (82.5–90.0)	87.3 (79.7–97.3)
Pulse Pressure, mmHg	39.3 (32.3–44.0)	45.3 (44.3–47.7) *	46.8 (42.3–49.1) *	48.0 (46.0–51.7) **	50.7 (46.0–55.1) **
Carotid-femoral PWV, m/s	8.47 (7.87–9.00)	8.97 (7.82–9.78)	9.75 (9.13–10.11) *	8.90 (8.63–10.09) *	11.27 (9.82–12.96) **
Carotid-radial PWV, m/s	9.21 (7.90–10.06)	9.76 (8.00–10.01)	9.58 (8.43–10.01)	9.83 (9.07–10.19)	11.17 (10.90–11.76) **
Nepalese Highlanders					
Parameters			3400 m a.s.l.	5050 m a.s.l.	
SaO_2_, %			94.0 (92.4–95.5)	85.0 (83.0–91.0) **^,†^	
Respiratory Rate, breaths/m			11.0 (11.0–12.0)	12.0 (12.0–13.0) ^†^	
Heart Rate, beat/m			62.7 (57.0–69.7)	64.3 (60.7–79.0)	
Systolic BP, mmHg			115.0 (109.7–118.7)	118.3 (102.7–121.3)	
Diastolic BP, mmHg			70.0 (66.7–76.0)	72.3 (70.7–78.7)	
Mean BP, mmHg			85.1 (80.8–90.2)	88.7 (81.6–92.4)	
Pulse Pressure, mmHg			42.7 (40.0–45.3)	42.7 (36.0–46.7) ^†^	
Carotid-femoral PWV, m/s			8.65 (7.73–9.97)	9.09 (8.59–11.17)	
Carotid-radial PWV, m/s			9.59 (8.54–11.64)	10.42 (8.92–12.21)	

Data are shown as median (interquartile range). Significance is expressed by the *p*-value. *, *p* < 0.05; **, *p* < 0.001; versus basal condition (sea level for lowlanders and 3400 m a.s.l. for highlanders). ^†^, *p* < 0.05; highlanders versus lowlanders at the same altitude (unadjusted data). BP, blood pressure; PWV, pulse wave velocity; SaO_2_, arterial oxygen saturation.

## Data Availability

The data presented in this study are available upon reasonable request from the corresponding author. The data are not publicly available due to privacy concerns.

## References

[1] Hackett P.H., Rennie D. (1978). Avoiding mountain sickness. Lancet.

[2] Selland M.A., Stelzner T.J., Stevens T., Mazzeo R.S., McCullough R.E., Reeves J.T. (1993). Pulmonary function and hypoxic ventilatory response in subjects susceptible to high-altitude pulmonary edema. Chest.

[3] Grocott M., Montgomery H., Vercueil A. (2007). High-altitude physiology and pathophysiology: Implications and relevance for intensive care medicine. Crit. Care.

[4] Hackett P.H., Rennie D., Levine H.D. (1976). The incidence, importance, and prophylaxis of acute mountain sickness. Lancet.

[5] Heistad D.D., Abboud F.M., Dickinson W. (1980). Richards Lecture: Circulatory adjustments to hypoxia. Circulation.

[6] Richalet J.P., Kacimi R., Antezana A.M. (1992). The control of cardiac chronotropic function in hypobaric hypoxia. Int. J. Sports Med..

[7] Rowell L.B., Blackmon J.R., Kenny M.A., Escourrou P. (1984). Splanchnic vasomotor and metabolic adjustments to hypoxia and exercise in humans. Am. J. Physiol..

[8] Milledge J.S., Catley D.M., Blume F.D., West J.B. (1983). Renin, angiotensin-converting enzyme, and aldosterone in humans on Mount Everest. J. Appl. Physiol. Respir. Environ. Exerc. Physiol..

[9] Aldashev A.A., Borbugulov U.M., Davletov B.A., Mirrakhimov M.M. (1989). Human adrenoceptor system response to the development of high altitude pulmonary arterial hypertension. J. Mol. Cell. Cardiol..

[10] Moore L.G. (2017). Human Genetic Adaptation to High Altitudes: Current Status and Future Prospects. Quat. Int..

[11] Brutsaert T.D., Soria R., Caceres E., Spielvogel H., Haas J.D. (1999). Effect of developmental and ancestral high altitude exposure on chest morphology and pulmonary function in Andean and European/North American natives. Am. J. Hum. Biol..

[12] Beall C.M., Brittenham G.M., Strohl K.P., Blangero J., Williams-Blangero S., Goldstein M.C., Decker M.J., Vargas E., Villena M., Soria R. (1998). Hemoglobin concentration of high-altitude Tibetans and Bolivian Aymara. Am. J. Phys. Anthropol..

[13] Lewis N.C., Bailey D.M., Dumanoir G.R., Messinger L., Lucas S.J., Cotter J.D., Donnelly J., McEneny J., Young I.S., Stembridge M. (2014). Conduit artery structure and function in lowlanders and native highlanders: Relationships with oxidative stress and role of sympathoexcitation. J. Physiol..

[14] Parati G., Revera M., Giuliano A., Faini A., Bilo G., Gregorini F., Lisi E., Salerno S., Lombardi C., Ramos Becerra C.G. (2013). Effects of acetazolamide on central blood pressure, peripheral blood pressure, and arterial distensibility at acute high altitude exposure. Eur. Heart J..

[15] Bollinger A., Hoffmann U., Franzeck U.K. (1991). Evaluation of flux motion in man by the laser Doppler technique. Blood Vessels.

[16] Salvi P. (2017). Pulse Waves. How Vascular Hemodynamics Affects Blood Pressure.

[17] Townsend R.R., Wilkinson I.B., Schiffrin E.L., Avolio A.P., Chirinos J.A., Cockcroft J.R., Heffernan K.S., Lakatta E.G., McEniery C.M., Mitchell G.F. (2015). Recommendations for Improving and Standardizing Vascular Research on Arterial Stiffness: A Scientific Statement from the American Heart Association. Hypertension.

[18] Nichols W., O’Rourke M., Vlachopoulos C. (2011). McDonald’s Blood Flow in Arteries. Theoretical, Experimental and Clinical Principles.

[19] Kelly R., Hayward C., Ganis J., Daley J., Avolio A., O’Rourke M. (1989). Non-invasive registration of the arterial pressure pulse waveform using highfidelity applanation tonometry. J. Vasc. Med. Biol..

[20] Reference Values for Arterial Stiffness Collaboration (2010). Determinants of pulse wave velocity in healthy people and in the presence of cardiovascular risk factors: ‘Establishing normal and reference values’. Eur. Heart J..

[21] Salvi P., Lio G., Labat C., Ricci E., Pannier B., Benetos A. (2004). Validation of a new non-invasive portable tonometer for determining arterial pressure wave and pulse wave velocity: The PulsePen device. J. Hypertens..

[22] Salvi P., Scalise F., Rovina M., Moretti F., Salvi L., Grillo A., Gao L., Baldi C., Faini A., Furlanis G. (2019). Noninvasive Estimation of Aortic Stiffness Through Different Approaches. Hypertension.

[23] Revera M., Salvi P., Faini A., Giuliano A., Gregorini F., Bilo G., Lombardi C., Mancia G., Agostoni P., Parati G. (2017). Renin-Angiotensin-Aldosterone System Is Not Involved in the Arterial Stiffening Induced by Acute and Prolonged Exposure to High Altitude. Hypertension.

[24] Parati G., Bilo G., Faini A., Bilo B., Revera M., Giuliano A., Lombardi C., Caldara G., Gregorini F., Styczkiewicz K. (2014). Changes in 24 h ambulatory blood pressure and effects of angiotensin II receptor blockade during acute and prolonged high-altitude exposure: A randomized clinical trial. Eur. Heart J..

[25] Bernardi L. (2007). Heart rate and cardiovascular variability at high altitude. Annu Int Conf IEEE Eng Med Biol Soc..

[26] Bernardi L., Passino C., Spadacini G., Calciati A., Robergs R., Greene R., Martignoni E., Anand I., Appenzeller O. (1998). Cardiovascular autonomic modulation and activity of carotid baroreceptors at altitude. Clin. Sci..

[27] Reinhart W.H., Kayser B., Singh A., Waber U., Oelz O., Bartsch P. (1991). Blood rheology in acute mountain sickness and high-altitude pulmonary edema. J. Appl. Physiol..

[28] Patel R.S., Al Mheid I., Morris A.A., Ahmed Y., Kavtaradze N., Ali S., Dabhadkar K., Brigham K., Hooper W.C., Alexander R.W. (2012). Oxidative stress is associated with impaired arterial elasticity. Atherosclerosis.

[29] Noma K., Goto C., Nishioka K., Jitsuiki D., Umemura T., Ueda K., Kimura M., Nakagawa K., Oshima T., Chayama K. (2007). Roles of rho-associated kinase and oxidative stress in the pathogenesis of aortic stiffness. J. Am. Coll. Cardiol..

[30] Hackett P.H., Rennie D. (1979). Rales, peripheral edema, retinal hemorrhage and acute mountain sickness. Am. J. Med..

[31] Bilo G., Caldara G., Styczkiewicz K., Revera M., Lombardi C., Giglio A., Zambon A., Corrao G., Faini A., Valentini M. (2011). Effects of selective and nonselective beta-blockade on 24-h ambulatory blood pressure under hypobaric hypoxia at altitude. J. Hypertens..

[32] Palombo C., Kozakova M., Morizzo C., Gnesi L., Barsotti M.C., Spontoni P., Massart F., Salvi P., Balbarini A., Saggese G. (2011). Circulating endothelial progenitor cells and large artery structure and function in young subjects with uncomplicated type 1 diabetes. Cardiovasc. Diabetol..

[33] Modesti P.A., Vanni S., Morabito M., Modesti A., Marchetta M., Gamberi T., Sofi F., Savia G., Mancia G., Gensini G.F. (2006). Role of endothelin-1 in exposure to high altitude: Acute Mountain Sickness and Endothelin-1 (ACME-1) study. Circulation.

[34] Bruno R.M., Cogo A., Ghiadoni L., Duo E., Pomidori L., Sharma R., Thapa G.B., Basnyat B., Bartesaghi M., Picano E. (2014). Cardiovascular function in healthy Himalayan high-altitude dwellers. Atherosclerosis.

[35] Parati G., Ochoa J.E., Torlasco C., Salvi P., Lombardi C., Bilo G. (2015). Aging, High Altitude, and Blood Pressure: A Complex Relationship. High Alt. Med. Biol..

